# Individual-Focused Approaches to the Prevention of College Student Drinking

**Published:** 2011

**Authors:** Jessica M. Cronce, Mary E. Larimer

**Keywords:** Alcohol consumption, problematic alcohol use, college students, prevention, college and university-based prevention, preventive intervention, individual-level prevention, brief motivational intervention, personalized feedback intervention, personalized normative feedback, blood alcohol concentration, prevention through education

## Abstract

Alcohol consumption is prevalent among college students and can become problematic for some. Numerous randomized controlled trials have evaluated the efficacy of individual preventive interventions in reducing alcohol use and alcohol-related problems in college student populations. Consistent with earlier reviews, the balance of the evidence from studies conducted during the past 3 years strongly supports the efficacy of brief motivational interventions combined with personalized feedback interventions (PFIs) and personalized normative feedback (PNF), as well as of stand-alone PFI/PNF interventions. Recent analyses also continue to support the efficacy of alcohol expectancy challenge interventions, although the findings are less consistent. In addition, recent analyses offer mixed support for feedback-based interventions focused solely on blood alcohol concentration and for multicomponent, alcohol education–focused interventions that include elements of PFI/PNF. No evidence of efficacy was found for programs that only included alcohol education.

As detailed by Johnston and colleagues (2009), the majority of young adults, in particular college students, consume alcohol. Moreover, a substantial proportion of those who consume alcohol misuse it, engaging in heavy episodic drinking,^1^ which directly and indirectly contributes to a host of harmful consequences (O’Malley and Johnston 2002; Perkins 2002). The rates of heavy drinking peak at ages 21 or 22 (Johnston et al. 2009), suggesting that most college students mature out of heavy drinking. Nevertheless, the harm they experience as a result of heavy drinking, such as poor academic and work performance or serious physical injury, may irrevocably alter students’ natural developmental trajectories. In an effort to prevent or mitigate such long-term harm, myriad prevention programs have been developed to reduce college student drinking by targeting individual factors associated with alcohol use and misuse, including alcohol expectancies, drinking motives, perceived norms, and natural ambivalence regarding behavior (Baer 2002; Presley et al. 2002). A wealth of research has been devoted to evaluating the efficacy of these preventive interventions. The purpose of this article is to provide a comprehensive summary of the current state of the science with regard to individual-focused preventive interventions whose efficacy in reducing alcohol use and alcohol-related problems has been evaluated in the college student population using randomized controlled trials. Conclusions from earlier reviews in this area are described briefly, with greater focus given to summarizing evidence accumulated in the past 3 years (2007–2010).

## Individual-Focused Preventive Interventions: Specific Components and Evidence of Efficacy

### Previous Reviews

Larimer and Cronce (2002, 2007) conducted qualitative reviews of research published between 1984 and early 2007 that evaluated the efficacy of individual preventive interventions aimed at college students. Both reviews noted a dearth of support for educational or awareness models, including information-based and values-clarification approaches, whereas there was evidence of efficacy for skills-based interventions, including self-monitoring/assessment, alcohol expectancy challenge (AEC), and multicomponent skills training. Moreover, both reviews documented strong empirical support for brief motivational interventions (BMIs) delivered via mail, online, or in person. As the name implies, in-person BMIs are brief (i.e., typically delivered over one or two sessions) and focus on enhancing motivation and commitment to change problematic behavior. To this end, BMIs often provide personalized feedback regarding the client’s drinking and related consequences, alcohol expectancies, and drinking motives; when delivered alone in the absence of a trained facilitator, this personalized feedback component is referred to as a personalized feedback intervention (PFI). BMIs and PFIs often additionally include general alcohol information (i.e., alcohol education) and alcohol-specific coping and harm-reduction skills. PFIs typically include personalized normative feedback (PNF), which compares the client’s self-reported drinking behavior to the average drinking behavior of a specific reference group (e.g., typical student, typical female). PNF encourages clients to explore and enhance discrepancies between their perception of their own drinking as “typical” and the actual drinking behaviors of their peers—that is, that the majority of students drink moderately, often significantly less than the individual receiving the intervention. Like PFIs, PNF can be delivered as a stand-alone intervention in the absence of in-person contact. Larimer and Cronce (2007) independently detailed empirical evidence supporting normative reeducation interventions, in particular computer-administered or in-person PNF interventions, that produced reductions in drinking and/or consequences mediated through changes in normative perceptions.

Complementing the qualitative reviews by Larimer and Cronce (2002, 2007), Carey and colleagues (2007) conducted a quantitative review evaluating 62 randomized clinical trials of 98 alcohol interventions for college students published during roughly the same time period (i.e., 1985 to early 2007). This meta-analysis similarly supported the efficacy of individual-focused alcohol interventions in reducing the quantity and frequency of alcohol use and alcohol-related negative consequences. The investigators further noted that significant intervention effects on indices of alcohol consumption peaked before the 6-month followup and that subsequently emerging effects on alcohol-related negative consequences lasted through long-term followup (ranging from 1 to 3.75 years). Specifically, Carey and colleagues (2007) concluded that individual interventions that used motivational interviewing techniques, included personalized feedback on alcohol expectancies and drinking motives with normative reeducation components, and included decisional balance exercises demonstrated greater efficacy in reducing alcohol-related consequences than did various comparison groups. This combination of intervention components is common to intervention approaches patterned after the Brief Alcohol Screening and Intervention for College Students (BASICS) program (Dimeff et al. 1999).

### Review of Recent Individual-Focused Preventive Intervention Studies

In the years since the publication of the reviews by Carey and colleagues (2007) and Larimer and Cronce (2002, 2007), numerous studies of individual-focused preventive interventions for college student drinking have been published. Of these, 36 studies evaluating 56 unique interventions, met criteria for inclusion in this review (see the tables for details). Studies were identified via a comprehensive search of electronic databases, including PsycINFO and MEDLINE (for a list of search terms used, see Larimer and Cronce 2007), covering the period from late 2007 to early 2010. Additional studies were identified indirectly (e.g., they were referenced in the introduction section of one of the identified studies), and as-yet-unpublished studies were provided directly by authors. Studies were included if they used a randomized controlled trial approach—that is, if they randomly assigned individual participants (or intact groups) to one of two or more experimental conditions, including at least one active intervention and an ostensibly inert control (e.g., assessment only) group. Although the number of studies meeting inclusion criteria suggests that a meta-analysis may be warranted, a qualitative approach was selected for this review to facilitate more rapid communication with key stakeholders concerning the current state of alcohol prevention.^2^ However, intervention effect sizes are reported for relevant outcomes in all studies that included effect size estimates in the original report or provided sufficient postintervention data to calculate between-group estimates (see tables). Within-group effect size estimates also are provided for studies wherein significant within-person reductions in alcohol use or consequences were evident.

Many of the studies included in this review evaluate the efficacy of multicomponent BMIs, many of which were adapted from the BASICS program. Most of these BMIs incorporated a PFI with PNF. Some studies evaluated one or more PFI/PNF interventions delivered alone, without the benefit of a trained intervention facilitator. Interventions were delivered via various modalities, including in-person group and individual sessions, mailed printed material, and Web-based content. In addition, some interventions were conducted in special settings (i.e., primary care, in the student’s home before entering college) or targeted high-risk student subpopulations (i.e., mandated/ sanctioned students, freshmen, or athletes).

#### Stand-Alone PFI/PNF Interventions

A total of 17 studies evaluated the impact of 14 unique PFIs/PNF and 4 PNF-only interventions implemented via written material, mail, computer, Web, or electronic diary on college student drinking (see table 1). Of 14 PFI/PNF interventions evaluated, 6 were associated with reductions in drinking but not drinking-related consequences relative to the comparison condition at followup. One PFI/PNF intervention (Doumas and Andersen 2009) was associated with reduced drinking-related consequences as well as alcohol use. Four additional PFI/PNF interventions were associated with significant within-person reductions in alcohol use and/or consequences across assessment periods, but between-group differences were not evident. Of four PNF-only interventions evaluated, three resulted in reductions in drinking outcomes at followup. The remaining PNF-only intervention had no effects on these outcomes but was associated with reductions in perceived drinking norms and increased readiness/ preparation for behavior change.

#### In-Person BMIs

The literature review also identified 17 studies evaluating 20 unique in-person BMIs (individual and group), most of which incorporated PFI and/or PNF (see table 2). Of these interventions, 13 were associated with reductions in drinking, alcohol-related negative consequences, and/or associated psychopathology, and three interventions exhibited a protective effect against the onset of or increase in alcohol use and/or related consequences. One of these studies (Schaus et al. 2009) demonstrated a sleeper effect of the intervention, with short-term reductions in drinking and subsequently emerging reductions in consequences. Also note that another of these studies (Doumas and Hannah 2008) was not specifically aimed at college students but targeted young adults (ages 18 to 24) who were employed; however, 75 percent of the sample concurrently was enrolled in school. This study found that BMI combined with PFI was equivalent to PFI alone in reducing drinking-related variables. Finally, one of these studies (Hansson et al. 2007) specifically evaluated intervention gains between the 12-month and 24-month followup and found an advantage for a BMI combined with coping skills over either component alone. A quantitative comparison of changes from baseline to the 12-month followup was not presented. However, figures displaying group means suggest a potential short- term effect of the BMI-only condition in reducing estimated blood alcohol concentrations (BACs), which, if counted, would bring the above total support for BMI conditions from 13 to 14.

Other conclusions that can be drawn from the analysis of these studies include the following:

- Findings of studies evaluating BMI in specialized settings and high-risk subpopulations suggest that primary care is an effective venue for delivery of this type of intervention (Schaus et al. 2009).
- Group BMI or BMI enhanced with parental coaching is effective in reducing drinking among college freshmen (Turrisi et al. 2009; Wood et al. 2010).
- BMI is effective for nonmandated high-risk drinkers (Doumas and Hannah 2008; Stahlbrandt et al. 2007).
- Studies involving students who had been mandated to participate in the interventions documented benefits of BMIs (Carey et al. 2009; White et al. 2007), in particular for females (Carey et al. 2009) and those who received additional services, including coping skills, problem solving, and stress management training, in the context of a student assistance program (Amaro et al. 2009). Another study (Carey et al. 2010) additionally found greater benefit of BMI participation in reducing alcohol consumption among female mandated students compared with two separate multicomponent educational programs; however, reductions in the BMI were similar to assessment only. Participation in any of the three interventions was associated with short-term reductions in alcohol consumption among male mandated students.

#### Other Preventive Approaches

Additional studies evaluated other specific alcohol interventions, in most cases comparing these approaches to other active interventions (e.g., BMI or PFI/PNF) (see table 3). Two studies published in the time period evaluated included alcohol expectancy challenge (AEC) protocols, which generally are considered to be more skills based than motivational in nature. Lau-Barraco and Dunn (2008) evaluated a single-session, gender-specific in vivo (experiential) AEC. In contrast, Wood and colleagues (2007) assessed a two-session mixed-gender in vivo AEC, both alone and in combination with a BMI involving a PFI/PNF component. Both AEC interventions resulted in reductions in alcohol use but not alcohol consequences.

Two other studies (Glindemann et al. 2007; Thombs et al. 2007) investigated the efficacy of BAC feedback, another cognitive–behavioral skills-based approach used to intervene with college students. One of these studies (Glindemann et al. 2007) demonstrated a positive effect of the intervention (i.e., reductions in BACs), whereas the other (Thombs et al. 2007) reported a potential inadvertent opposite (i.e., iatrogenic) effect—that is, an increase in BACs. These mixed findings may be related to differences between the two studies in terms of the timing of the feedback (i.e., immediate versus delayed) and use of incentives to promote lower BACs (i.e., a $100 cash raffle for participants with BACs lower than 0.05 percent in the study by Glindemann and colleagues [2007]).

Four studies evaluated alcohol education either as a stand-alone intervention (see Thadani et al. 2009) or as a comparison intervention for PFI/PNF interventions with or without BMI. These studies generally found increases in alcohol knowledge among the students receiving the intervention. However, the interventions generated equivocal or negative effects on alcohol use and related consequences because they detected no group differences and/or lacked an assessment-only control group.

Finally, eight studies tested nine unique multicomponent, education-focused programs, which included general alcohol information as well as elements typically associated with efficacious BMI and PFI/PNF interventions, such as personalized feedback, normative reeducation, challenge of positive drinking expectancies, and tips for harm reduction. Just over one-half of these programs were associated with reductions in drinking and/or alcohol consequences, whereas the remainder (i.e., Alcohol 101 Plus [Carey et al. 2009]; an in-person, facilitator-led program [Cimini et al. 2009]; AlcoholEdu for College, 2006 version [Croom et al. 2008]; and Alcohol 101 [Lau-Barraco and Dunn 2008]) produced equivocal results. Of note, because the effective multicomponent education programs (e.g., AlcoholEdu, 2007 version; AlcoholEdu for College; AlcoholEdu for Sanctions; and College Alc) included BMI and PFI/PNF elements, it is impossible to disentangle the effect of education alone from the effects of these efficacious components.

## Individual-Focused Preventive Interventions: Conclusions and Future Research

In summary, studies published between 2007 and early 2010 provide consistent support for the efficacy of brief, personalized, individual motivational feedback (i.e., BMI with PFI/PNF) interventions and stand-alone PFI/PNF interventions. These studies also provide support for the efficacy of AEC interventions, although less consistent, and offer mixed support for BAC feedback. These conclusions are in line with previous reviews (Carey et al. 2007; Larimer and Cronce 2002, 2007). Also consistent with previous reviews, there was an absence of support for programs solely including alcohol education, although multicomponent alcohol education–focused programs, which combine educational elements with BMI, PFI, and PNF components, had greater, albeit mixed, support.

Although the balance of the evidence supports the efficacy of PFI/PNF-only interventions, additional research on these interventions is necessary to identify the elements and/or modalities that are associated with behavior change and to determine for whom in-person BMI is more (or less) efficacious compared with PFI/PNF-only interventions. The lack of intervention effects in a few of the BMI and PFI/PNF studies may reflect the potential absence (or ineffective delivery) of necessary intervention components or the presence of potential moderators of intervention effects (e.g., mandated student status). Additional research also needs to establish the efficacy of these brief interventions in reducing long-term risk. Thus, it may be necessary to modify and evaluate existing interventions and/or evaluate the effects of supplemental interventions in order to extend their short-term effects and enhance or prolong their impact on negative drinking consequences. Recent findings (Carey et al. 2007; Schaus et al. 2009) suggesting longer-term emergent effects on alcohol-related consequences, particularly in response to in-person BMIs (Carey et al. 2007), indicate that the addition of longer-term follow-up assessments will be necessary to achieve this. Finally, additional research is needed to evaluate the efficacy of BMIs in combination with other interventions, including interventions targeting environmental change, parenting practices, or psychiatric comorbidity. Ultimately, multiple intervention strategies may be necessary to produce lasting effects on college student drinking and related harm.

Unfortunately, key stakeholders (e.g., college administrators, campus health professionals) face numerous barriers when trying to implement efficacious individual-focused alcohol interventions. For example, with the exception of commercially available programs, such as e-Chug or AlcoholEdu, the measures and feedback programs used in most intervention protocols are not easily accessible or not immediately useable. For those seeking to implement the BASICS approach (Dimeff et al. 1999), a published manual and measures are available. However, campus personnel may not have adequate resources (e.g., the expertise to train and supervise therapists, access to programs that can generate personalized feedback, or access to campus specific normative drinking data) to implement the program with sufficient fidelity.

Many of these barriers can be overcome by pairing health and counseling personnel with faculty in academic departments who may have experience with program evaluation and implementation. Word processing and spreadsheet/database programs generally available to campus personnel can be used to generate basic personalized feedback. Distance-learning methods currently used to disseminate some evidence-based public health interventions (e.g., video- or Web-based conferencing of initial training and ongoing clinical supervision) could be adapted to support implementation of BMI protocols. Implementation of routine alcohol screening in campus health centers could be used to gather normative data for use in PFI/PNF and to identify students appropriate for intervention.

Barriers to intervention implementation also necessitate additional research into increasing the reach of evidence-based approaches. This includes research related to training of providers and assessment of fidelity for in-person interventions, methods to improve impact and portability of Web-based or mailed/written interventions, and research on adaptation of efficacious interventions so they are appropriate for young adults from different cultural backgrounds and in contexts outside the traditional, mainstream college setting. To date, young adults in the workplace, community-college settings, tribal colleges and universities, historically Black colleges and universities, and other minority-serving institutions have been substantially underrepresented in efficacy trials of BMIs and related interventions. Careful consideration and the development of meaningful community partnerships to support the bidirectional learning necessary to adapt and implement efficacious brief prevention approaches in these settings are needed.

## Figures and Tables

**Table 1:** Studies Assessing the Efficacy of Stand-Alone PFI/PNF Interventions Compared With Assessment Only or Other Interventions

**Study**	**Intervention Conditions**	**Student Population Outcome (Intervention Condition)**	**Effect Sizes**	**Follow-up Period**
**PFI/PNF vs. assessment only**				
Bewick et al. (2008)	1. Web-based PFI/PNF*	Reduced drinks per drinking occasion (1)	*d* = 0.29	12 weeks
	2. Assessment only			
Doumas & Andersen (2009)	1. Web-based PFI/PNF (e-Chug)*	*Among high-risk drinkers:*		3 months
		Reduced frequency of intoxication (1)	*d* = 0.85	
	2. Assessment only	Reduced alcohol consequences (1)	*d* = 0.80	
Geisner, et al. (2007)	1. Mailed PFI/PNF with general tips	Reduced perceived drinking norms (1)	*d* = 0.60	1 month
		No group difference with respect to alcohol use or consequences (1, 2)		
	2. Assessment only			
Hustad et al. (2010)	1. Web-based PFI/PNF (e-Chug)*	Reduced typical and peak drinking (1)	*d* s = 0.54 to 0.85	1 month
		Reduced typical and peak drinking (2)	*d* s = 0.59 to 0.75	
	2. Multicomponent alcohol education–focused program (AlcoholEdu)	Reduced alcohol consequences (2)	*d* = 0.56	
	3. Assessment only			
Weitzel et al. (2007)	1. PFI/PNF only	Reduced drinks per drinking day during the intervention period, but not at followup (1)	N/A	2 weeks
	2. Assessment only			
**PFI/PNF vs. waitlist control**				
White et al. (2008)	1. PFI/PNF (within person*)	*Mandated/sanctioned students:*		2 months and 7 months
	2. Waitlist control (received PFI with PNF based on baseline assessment at first followup) (within person*)	No group differences (1, 2)		
		*Within-person comparisons:*	*Within-person d* s :	
		Reduced drinking frequency (1, 2)	*d* s = 0.23, 0.28	2 months
		Reduced heavy drinking episodes (1)	*d* = 0.29	
		Reduced peak BAC (1, 2)	*d* s = 0.24, 0.28	
		Reduced alcohol consequences (2)	*d* = 0.23	
		Reduced drinking frequency (1, 2)	*d* s = 0.24, 0.28	7 months
		Reduced peak BAC (2)	*d* = 0.22	
		Reduced alcohol consequences (1, 2)	*d* s = 0.20, 0.19	
**PFI/PNF vs. alcohol education**				
Doumas & Haustveit (2008)	1. Web-based PFI/PNF*	*Among high-risk drinkers:*	ηp^2^= 0.14	6 weeks and 3 months
	2. Alcohol education	Reduced weekly drinking quantity (1)	ηp^2^= 0.15	
		Reduced peak drinking quantity (1)	ηp^2^= 0.20	
		Reduced frequency of intoxication (1)		
		Drinking reductions were positively associated with reductions in perceived norms for typical student drinking		
Doumas et al. (2009)	1. Web-based PFI/PNF*	*Mandated/sanctioned students:*	ηp^2^= 0.07	30 days
	2. Web-based alcohol education (Judicial Educator)	Reduced weekly drinking quantity (1)	ηp^2^= 0.08	
		Reduced peak drinking quantity (1)	ηp^2^= 0.07	
		Reduced frequency of intoxication (1)		
		Changes in drinking were mediated via reductions in perceived norms for alcohol consumption		
**Minimal PFI/PNF vs. enhanced PFI/PNF**				
Saitz et al. (2007)	1. Minimal Web-based PFI/PNF (within person*)	*High-risk drinking freshmen:*		
		No group differences (1, 2)		
		*Within-person comparisons:*		1 month
	2. Enhanced Web-based PFI/PNF (within person*)	Reduced AUDIT scores (1, 2)		
		Reduced quantity drinks per week (women; 1, 2)		
		Reduced heavy drinking episodes (women; 1, 2)		
**PFI/PNF vs. BMI**	1. In-person BMI with PFI	Reduced frequency of typical drinking (1, 2)	ηp^2^ = 0.13	4 weeks
Butler et al. (2009)	2. Computerized PFI alone*	Reduced quantity of typical drinking (1, 2)	ηp^2^ = 0.17	
	3. Assessment only	Reduced frequency of binge drinking (1, 2)	ηp^2^ = 0.15	
Doumas & Hannah (2008)	1. BMI with Web-based PFI/PNF	*Among high-risk drinkers:*	ηp^2^ = 0.07	30 days
		Reduced weekend alcohol use (1, 2)	ηp^2^ = 0.05	
	2. Web-based PFI/PNF only*	Reduced peak drinking quantity (1, 2)	ηp^2^ = 0.04	
		Reduced frequency of intoxication (1, 2)		
	3. Assessment only			
Mun et al. (2009)	1. BMI with PFI/PNF	No group differences (1, 2)		15 months
	2. Written PFI/PNF only			
Walters et al. (2009)	1. BMI with PFI/PNF	Reduced alcohol use and problems (1)	*d* = 0.54	6 months
	2. BMI without PFI/PNF	No group differences on alcohol use or consequences (2, 3, 4)		
	3. Web-based PFI/PNF only			
	4. Assessment only			
White et al. (2007)	1. BMI with PFI/PNF	*Mandated/sanctioned students:*	N/A	4 months
	2. Written PFI/PNF only (within person*)	No group differences (1, 2)	*d* = 0.27	15 months
		Protective effect against increases in alcohol consequences (1)		
		*Within-person comparisons:*	*Within-person d*s:	15 months
		Reduced quantity drinks per week (1)	*d* = 0.28	
		Reduced peak BAC (1, 2)	*d* = 0.36, 0.19	
		Reduced alcohol consequences (1)	*d* = 0.39	
**PNF-only vs. assessment only**				
Lewis et al. (2007	1. Gender-specific computerized PNF*	Reduced quantity drinks per week (1)	N/A	5 months
		Reduced drinking frequency (1)		
	2. Gender-neutral computerized PNF*	Reduced drinking frequency (2)		
	3. Assessment only			
Lewis et al. (2008)	1. 21st birthday card with PNF	Reduced normative misperceptions (1)	ηp^2^ = 0.07	1 -week
		No group differences with respect to alcohol use or consequences (1,2)		
	2. Assessment only			
Neighbors et al. (2009)	1. 21st birthday card with PNF*	Reduced BAC on 21st birthday (1)	*d* = 0.33	4 days post-birthday
		Intervention was more effective among those with baseline intentions to reach higher BACs		
	2. Assessment only			

NOTE: Mun et al. (2009) reported the outcome of subsequent analyses related to the efficacy of interventions originally reported in White et al. (2007); as such, these interventions are not included in the total count of unique interventions provided in the text.

Intervention conditions followed by an “*” indicates the specific intervention was associated with reductions, or exhibited a protective effect against, relevant behavioral outcomes (e.g., quantity or frequency of alcohol consumption; alcohol-related negative consequences). Effect sizes reported include Cohen's *d* (Cohen, 1988), which denotes the standardized difference between the mean of the intervention and comparisons groups and eta squared (η2), which denotes the proportion of total variability in the dependent variable attributable to the effect of the independent variable, or partial eta squared (ηp2). According to Cohen's (1988, 1992) definitions of effect size, small, medium, and large effects for *d* are considered to be in the 0.20, 0.50, and 0.80 ranges, respectively, and for η2 and ηp2 are 0.01, 0.06, and 0.14, respectively. N/A = effect size estimate not available.

**Table 2:** Studies Assessing the Efficacy of In-Person BMIs

**Study**	**Intervention Conditions**	**Student Population Outcome (Intervention Condition)**	**Effect Sizes**	**Follow-up Period**
**BMI vs. assessment only**				
Amaro et al. (2009)	1. In-person BMI with PFI plus indicated cognitive–behavioral interventions*	*Mandated/sanctioned students:*		
		Reduced weekday alcohol use (1)	*d* = 1.06	6 months
		Reduced alcohol consequences (1)	*d* = 0.65	
	2. Counseling services as usual	Increased use of protective behavioral strategies (1)	*d* = 1.98	10 weeks
LaBrie et al. (2008)	1. Group BMI*	*Freshmen women:*		
	2. Assessment only	Reduced typical drinking (1)	*d* = 0.34	
		Reduced heavy-episodic drinking (1)	*d* = 0.42	
		Intervention was more effective for those with higher social and enhancement drinking motives		
LaBrie et al. (2009)	1. Group BMI	*Freshmen women:*		6 months
	2. Assessment only	No group differences (1, 2)		
**BMI vs. PFI/PNF only**				
Butler et al. (2009)	1. In-person BMI with PFI*	Reduced frequency of typical drinking (1, 2)	ηp^2^ = 0.13	4 weeks
		Reduced quantity of typical drinking (1, 2)	ηp^2^= 0.17	
	2. Computerized PFI alone	Reduced frequency of binge drinking (1, 2)	ηp^2^ = 0.15	
	3. Assessment only			
Doumas & Hannah (2008)	1. BMI with Web-based PFI/PNF*	*Among high-risk drinkers:*		
		Reduced weekend alcohol use (1, 2)	ηp^2^ = 0.07	30 days
	2. Web-based PFI/PNF only	Reduced peak drinking quantity (1, 2)	ηp^2^ = 0.05	
		Reduced frequency of intoxication (1, 2)	ηp^2^ = 0.04	
	3. Assessment only			
Mun et al. (2009)	1. BMI with PFI/PNF	No group differences (1, 2)		15 months
	2. Written PFI/PNF only			
Walters et al. (2009)	1. BMI with PFI/PNF*	Reduced alcohol use and problems (1)	*d* = 0.54	6 months
	2. BMI without PFI/PNF	No group differences on alcohol use or consequences (2, 3, 4)		
	3. Web-based PFI/PNF only			
	4. Assessment only			
White et al. (2007)	1. BMI with PFI/PNF*	*Mandated/sanctioned students:*		4 months
	2. Written PFI/PNF only	No group differences (1, 2)	N/A	15 months
		Protective effect against increases in alcohol consequences (1)	*d* = 0.27	
		*Within-person comparisons:*	*Within-person d*s :	
		Reduced quantity drinks per week (1)	*d* = 0.28	15 months
		Reduced peak BAC (1, 2)	*d* = 0.36, 0.19	
		Reduced alcohol consequences (1)	*d* = 0.39	
**BMI vs. other interventions**				
Carey et al. (2009)	1. In-person BMI with PNF*	*Mandated/sanctioned students:*		
	2. Multicomponent alcohol education-focused program (*Alcohol 101 Plus*)	Reduced alcohol use (various indices) among women only (1)	*d*s = 0.21 to 0.38	1 month
Carey et al. (2010)	1. In-person BMI with PFI/PNF*	*Mandated/sanctioned students:*	N/A	1 month
		Reduced alcohol use (various indices) among men (1, 2, 3)		
	2. Multicomponent alcohol education–focused program (*Alcohol 101 Plus*)			
		No group differences on problems among men (1, 2, 3, 4)		
	3. Multicomponent alcohol education–focused program (*AlcoholEdu for Sanctions*)	Reduced alcohol use *without* group differences among women(1, 2, 3, 4)		
		Reduced problems *without* group differences among women (1, 3, 4)		
	4. Waitlist control	Women in (1) experienced greater reductions in alcohol use relative to (2, 3)		
Cimini et al. (2009)	1. Group BMI	*Mandated/sanctioned students:*		6 months
	2. Interactive peer theatrical presentation	No group differences (1, 2, 3)		
	3. In-person alcohol education			
Hansson et al. (2007)	1. BMI(possible *; refer to article)	Reduced alcohol psychopathology (3)	*d*s = 0.52 to 0.60	12–24 months
		Reduced drinking consequence scores (3)	*d*s= 0.42 to 0.72	
	2. Coping skills training	Reduced estimated BACs (3)	*d* = 0.49	
	3. BMI + coping skills training*			
Schaus et al. (2009)	1. BMI with PNF*	Reduced typical drinking (1)	*d*s = 0.27–0.41	3 and 6 months
	2. Alcohol education	Reduced peak drinking (1)	*d*s = 0.25–0.36	
		Reduced typical BAC (1)	*d*s = 0.28–0.35	
		Reduced peak BAC (1)	*d*s = 0.37–0.49	
		Reduced frequency of intoxication (1)	*d*s = 0.42–0.50	
		Reduced alcohol problems (1)	*d*s = 0.23–0.29	6 and 9 months
Stahlbrandt et al. (2007)	1. Modified group BASICS-based BMI*	*Among high-risk drinkers:*	*d* = 0.27	2 years
		Reduced AUDIT scores (1)		
	2. 12-step focused group			
	3. Assessment only			
Turrisi et al. (2009)	1. Parent-based intervention (PMI)	Reduced typical drinking (3)	*d*s = 0.14–0.20	10 months
		Reduced peak drinking (3)	*d*s = 0.17–0.26	
	2. BMI with PFI/PNF*	Reduced alcohol consequences (3)	*d*s = 0.13–0.20	
	3. PMI + BMI*	Changes in drinking were mediated via reductions in perceived descriptive and injunctive norms for alcohol consumption		
	4. Assessment only			
		Reduced peak BAC (2)	*d* = 0.16	
		Reduced number of drinks/weekend (2)	*d*s = 0.16–0.18	
Wood et al. (2007)	1. BMI with PFI/PNF*	Reduced total alcohol use (1)	*d*s = 0.16–0.25	1 month, 3 months, and 6 months
	2. Alcohol expectancy challenge (AEC)	Reduced total alcohol use (2)	*d*s = 0.01–0.20	
		Reduced heavy episodic consumption (1)	*d*s = 0.18–0.26	
	3. BMI with PFI/PNF + AEC	Reduced heavy episodic consumption (2)	*d*s = 0.00–0.22	
	4. Assessment only	Reduced alcohol consequences (1)	*d*s = 0.29–0.33	
Wood et al. (2010)	1. BMI with PFI/PNF*	*Protective effect against:*		
	2. Parent-based intervention (PMI)	Initiating heavy episodic consumption (1)	*h*s = 0.02–0.22	10 months and 22 months
		Experiencing onset alcohol consequences (1)	*h*s = 0.07–0.15	
	3. BMI + PMI*	Experiencing onset alcohol consequences (3)	N/A	
	4. Assessment only			

NOTE: conditions followed by an “*” indicates the specific intervention was associated with reductions, or exhibited a protective effect against, relevant behavioral outcomes (e.g., quantity or frequency of alcohol consumption; alcohol-related negative consequences). Mun et al. (2009) and LaBrie et al. (2009) both reported the outcome of subsequent analyses related to the efficacy of interventions originally reported in White et al. (2007) and LaBrie et al. (2008), respectively; as such, these interventions are not included in the total count provided in the text. Effect sizes reported include Cohen's *d* (Cohen, 1988), which denotes the standardized difference between the mean of the intervention and comparisons groups, Cohen’s *h* (Cohen, 1988), which denotes the difference between two proportions, and eta squared (ηp2), which denotes the proportion of total variability in the dependent variable attributable to the effect of the independent variable, or partial eta squared (ηp2). According to Cohen's (1988, 1992) definitions of effect size, small, medium, and large effects for *d* and *h* are considered to be in the 0.20, 0.50, and 0.80 ranges, respectively, and for η2 and ηp2 are 0.01, 0.06, and 0.14, respectively. N/A = effect size estimate not available.

**Table 3:** Studies Assessing the Efficacy of Other Preventive Interventions

**Study**	**Intervention Conditions**	**Student Population Outcome (Intervention Condition)**	**Effect Sizes**	**Follow-up Period**
**Alcohol expectancy challenge**				
Lau-Barraco and Dunn (2008)	1. Alcohol expectancy challenge (AEC)*	Reduced quantity of drinks per week (1)	*d*s = 0.30 to 0.35	1 month
		Reduced frequency of binge drinking (1)	*d*s = 0.34 to 0.36	
	2. Multicomponent alcohol education–focused program (Alcohol 101)			
	3. Assessment only			
Wood et al. (2007)	1. BMI with PFI/PNF	Reduced total alcohol use (1)	*d*s = 0.16–0.25	1 month, 3 months, and 6 months
	2. Alcohol expectancy challenge (AEC)*	Reduced total alcohol use (2)	*d*s = 0.01–0.20	
		Reduced heavy episodic consumption (1)	*d*s = 0.18–0.26	
	3. BMI with PFI/PNF + AEC	Reduced heavy episodic consumption (2)	*d*s = 0.00–0.22	
	4. Assessment only	Reduced alcohol consequences (1)	*d*s = 0.29–0.33	
**Blood alcohol concentration (BAC) feedback**				
Glindemann et al. (2007)	1. BAC feedback*	Lower BACs (1)	*d* = 0.31	Unspecified
	2. Assessment only	Increased percentage of individuals with a BAC <.08 g % (1)	*d* = 0.20	
Thombs et al. (2007)	1. BAC feedback	Increased observed mean BAC (2)	*d* = 0.30	Next day follow-up, aggregated across participants over 2-year project period
	2. BAC feedback + normative re-education			
**Alcohol education**				
Doumas & Haustveit (2008)	1. Web-based PFI with PNF	*Among high-risk drinkers:*	ηp^2^ = 0.14	6 weeks and
	2. Alcohol education	Reduced weekly drinking quantity (1)	ηp^2^ = 0.15	3 months
		Reduced peak drinking quantity (1)	ηp^2^ = 0.20	
		Reduced frequency of intoxication (1)		
		Drinking reductions were positively associated with reductions in perceived norms for typical student drinking		
Doumas et al. (2009)	1. Web-based PFI with PNF	*Mandated/sanctioned students:*	ηp^2^ = 0.07	30 days
	2. Internet-based alcohol education (Judicial Educator)	Reduced weekly drinking quantity (1)	ηp^2^ = 0.08	
		Reduced peak drinking quantity (1)	ηp^2^ = 0.07	
		Reduced frequency of intoxication (1) Changes in drinking were mediated via reductions in perceived norms for alcohol consumption		
		Schaus et al. (2009)	1. BMI with PNF	Reduced typical drinking (1)	*d*s = 0.27–0.41	3 months and
			2. Alcohol education	Reduced peak drinking (1)	*d*s = 0.25–0.36	6 months
				Reduced typical BAC (1)	*d*s = 0.28–0.35	
Reduced peak BAC (1)	*d*s = 0.37–0.49			
Reduced frequency of intoxication (1)	*d*s = 0.42–0.50			
Reduced alcohol problems (1)	*d*s = 0.28–0.29			6 months and 9 months
Thadani et al. (2009)	1. Alcohol education	*Freshmen women:*	*d* = 0.73	6 months
	2. Assessment only	Increased alcohol knowledge (1)		
		No group differences on alcohol use or consequences (1,2)		
**Multicomponent, education-focused interventions**				
Bersamin et al. (2007)	1. Multicomponent alcohol education–focused program (College Alc)*	*Freshmen:*		3 months
		Reduced heavy episodic consumption (1)	*d* = 0.15	
	2. Assessment only			
Carey et al. (2009)	1. In-person BMI with PNF	*Mandated/sanctioned students:*		
	2. Multicomponent alcohol education–focused program (Alcohol 101 Plus)	Reduced alcohol use (various indices) among women only (1)	*d* s = 0.21 to 0.38	1 month
Carey et al. (2010)	1. In-person BMI with PFI/PNF	*Mandated/sanctioned students:*		
		Reduced alcohol use (various indices)	NA	1 month
	2. Multicomponent alcohol education–focused program (Alcohol 101 Plus)*	among men (1, 2, 3)		
		No group differences on problems among men (1, 2, 3, 4)		
	3. Multicomponent alcohol education-focused program (AlcoholEdu for Sanctions)*	Reduced alcohol use *without* group differences among women (1, 2, 3, 4)		
		Reduced problems *without* group differences among women (1, 3, 4)		
	4. Waitlist control	Women in (1) experienced greater reductions in alcohol use relative to (2, 3)		
Cimini et al. (2009)	1. Group BMI	*Mandated/sanctioned students:*		6 months
	2. Interactive peer theatrical presentation	No group differences (1, 2, 3)		
	3. In-person multicomponent alcohol education–focused program			
Croom et al. (2008)	1. Multicomponent alcohol education–focused program (*AlcoholEdu for College*)	Increased alcohol knowledge (1)	*d* = 0.52	6 weeks’ post-
		Lower participation in drinking games (1)	*d* = 0.12	matriculation
		*Less* likely to use safer sex strategies (1)	N/A	
		No group differences with respect to alcohol use or consequences (1, 2)		
	2. Assessment only			
Hustad et al. (2010)	1. Web-based PFI with PNF (*e-Chug*)	Reduced typical and peak drinking (1)	*d* s = 0.54 to 0.85	1 month
		Reduced typical and peak drinking (2)	*d* s = 0.59 to 0.75	
	2. Multicomponent alcohol education–focused program (*AlcoholEdu for College*)*	Reduced alcohol consequences (2)	*d* = 0.56	
	3. Assessment only			
Lau-Barraco and Dunn (2008)	1. Alcohol expectancy challenge (AEC)			
		Reduced quantity of drinks per week (1)	*d* s = 0.30 to 0.35	1 month
	2. Multicomponent alcohol education–focused program (*Alcohol 101*)	Reduced frequency of binge drinking (1)	*d* s = 0.34 to 0.36	
	3. Assessment only			
Lovecchio et al. (2010)	1. Multicomponent alcohol education–focused program (*AlcoholEdu*)*	Increased alcohol knowledge (1)	*d* = 0.11	1 month
		*Decreased* responsible drinking behavior (1)	*d* = 0.28	
		*Protective effect against:*		
	2. Assessment only	Increased alcohol consequences (1)	*d* = 0.59	
		Increased accepting others’ drinks (1)	*d* = 0.65	
		Increased positive alcohol expectancies (1)	*d* = 0.07	

NOTE: conditions followed by an “*” indicates the specific intervention was associated with reductions, or exhibited a protective effect against, relevant behavioral outcomes (e.g., quantity or frequency of alcohol consumption; alcohol-related negative consequences). Effect sizes reported include Cohen's *d* (Cohen, 1988), which denotes the standardized difference between the mean of the intervention and comparisons groups, Cohen’s *h* (Cohen 1988), which denotes the difference between two proportions, and eta squared (η2), which denotes the proportion of total variability in the dependent variable attributable to the effect of the independent variable, or partial eta squared (ηp2). According to Cohen's (1988, 1992) definitions of effect size, small, medium, and large effects for d and h are considered to be in the 0.20, 0.50, and 0.80 ranges, respectively, and for η2 and ηp2 are 0.01, 0.06, and 0.14, respectively. NA = effect size estimate not available.

## Footnotes

1 The National Institute on Alcohol Abuse and Alcoholism (NIAAA) defines binge or heavy episodic drinking as the consumption of an amount of alcohol leading to a blood alcohol concentration (BAC) of 0.08 percent, which, for most adults, would be reached by consuming five drinks for men or four for women over a 2-hour period (NIAAA 2004). Wechsler and colleagues (1995) similarly denote a binge episode as consumption of five or more drinks for men and four or more drinks for women but do not stipulate a bounded time frame during which consumption must occur or link the episode to a particular BAC. The latter definition by Wechsler and colleagues (1995) was used most frequently across the studies reviewed here.

2 Both meta-analytic (quantitative) and qualitative reviews seek to combine findings from multiple studies addressing a shared research hypothesis (e.g., that a particular type of intervention will reduce alcohol use and/or consequences). In a meta-analysis, findings are combined via a common measure of effect size (e.g., Cohen’s *d*), and conclusions are based on a weighted average of all of the effect sizes. By comparison, a qualitative approach is more inductive, and conclusions summarize the balance of the evidence based on an additive evaluation of the separate studies.
